# A mixed-methods evaluation of community-based healthy kitchens as social enterprises for refugee women

**DOI:** 10.1186/s12889-019-7950-3

**Published:** 2019-11-29

**Authors:** Nadine R. Sahyoun, Zeina Jamaluddine, Jowel Choufani, Sandra Mesmar, Amelia Reese-Masterson, Hala Ghattas

**Affiliations:** 1 Department of Nutrition and Food Science, University of Maryland, College Park, MD USA; 2 Center for Research on Population and Health, Faculty of Health Sciences, American University of Beirut, POBox 11-0236/EPHD, Riad El-Solh, Beirut, 1107 2020 Lebanon; 3 International Food Policy Research Institute, Washington DC, USA; 4 CitySeed, Inc., New Haven, CT USA

**Keywords:** Community kitchens, Decision making, Food security, Refugees, Social enterprise

## Abstract

**Background:**

The aim of this study is to investigate the potential impact of a community-based intervention - the Healthy Kitchens, Healthy Children (HKHC) intervention - on participating women’s household’s economics and food security status, decision making, mental health and social support**.**

**Methods:**

We established two healthy kitchens in existing community-based organizations in Palestinian camps in Lebanon. These were set up as small business enterprises, using participatory approaches to develop recipes and train women in food preparation, food safety and entrepreneurship. We used a mixed-methods approach to assess the impact of participating in the program on women’s economic, food security, decision making, social and mental health outcomes. A questionnaire was administered to women at baseline and at an 8-month endpoint. The end line survey was complemented by a set of embedded open-ended questions.

**Results:**

Thirty-two Palestinian refugee women were employed within the kitchens on a rotating basis. Participating women had a 13% increase in household expenditure. This was translated into a significant increase in food (*p* < 0.05) and clothing expenditures (*p* < 0.01), as well as a reduction in food insecurity score (*p* < 0.01). These findings were supported by qualitative data which found that the kitchens provided women with financial support in addition to a space to form social bonds, discuss personal issues and share experiences.

**Conclusions:**

This model created a social enterprise using the concept of community kitchens linked to schools and allowed women to significantly contribute to household expenditure and improve their food security.

## Background

The Palestinian refugee presence in Lebanon dates back to 1948, with the majority of refugees living in urban camps with deteriorating infrastructure. In Lebanon, Palestinians face social and political exclusion including restrictions on employment [1], and have fragile livelihoods and high rates of poverty. Almost two thirds of the population lives below the poverty line and severity of household food insecurity is highly sensitive to changes in household income [1]. Female-headed households are particularly at risk of food insecurity as women in this population experience high rates of unemployment [1]. Female labor force participation rate is also low at 17%, likely related both to gender-norms and low education levels [2, 3]. In addition, food insecurity has been shown to negatively affect dietary diversity as well as physical, mental and social health in Palestinian refugees living in Lebanon [4].

One intervention with promise to address these issues is participation in community kitchens. Community kitchens are defined as community-based cooking programs which aim to enhance food preparation skills [5]. Most commonly, participants in community kitchens are trained in budgeting, menu planning, food hygiene, cooking skills, and may also receive nutrition education. Community kitchens involve regular meetings of participants to prepare meals which are then shared. The main differences between community kitchens and other food assistance programs are their collaborative, participatory aspects and their potential to foster social skills and social support [5]. Evidence for the impact of community kitchens comes almost exclusively from interventions implemented for low-income communities in high-income countries (Canada, Australia, Scotland), and highlights their role in increasing self-efficacy, social engagement, access to employment, and mental health [6–8]. A systematic review showed that by building a safe environment and decreasing social isolation, community kitchens enhance social interaction [7].

Findings regarding the effects of community kitchens on food security and nutritional status are however, less conclusive. Several studies report improvements in knowledge about nutrition, healthy food purchases and practices as well as increased dietary diversity [6, 9–11]. One study showed that participating in community kitchens was associated with improved short-term food security, decreased food security-related psychological stress, and increased awareness of food-related issues [12]. However, this study and others cite the need for further investigation of the effects of community kitchens on long-term food security, particularly as community kitchens do not significantly change the economic status of households, and thus have limited capacity to improve food security status [10, 12, 13]. Two reviews of the impact of community kitchens have concluded that there is insufficient evidence regarding whether community kitchens can address long-term resource-related food insecurity [7, 14]; this is likely due to the lack of an income-generating component in the models that have previously been implemented.

Integrating a livelihood-generating component into community kitchen interventions thus has the potential to tackle this shortfall, and impact food security status. In this study, we evaluated the potential of such a model in a refugee population living in a middle-income context, and investigated changes in household economics and food security status, decision making, mental health and social support in participating women.

## Methods

### Study context and intervention description

The UN Relief and Works Agency for Palestine refugees (UNRWA) has been providing assistance and protection to registered Palestinian refugees in Lebanon since 1950. Inside the urban camps where 63% of Palestinians refugees in Lebanon reside, UNRWA provides infrastructure (water, electricity, housing) in addition to education, health care and other welfare services to eligible refugees [1]. In this context, we designed the *Healthy Kitchens, Healthy Children (HKHC)* intervention to address long-term food insecurity in two UNRWA camps in Beirut, Lebanon. Two community kitchens were established as small business enterprises for Palestinian women and were linked to two UNRWA elementary schools to prepare and cater healthy snacks to school children for the duration of one academic year. The intervention took place in two camps (Bourj el Barajneh and Shatila). We worked with UNRWA’s social services program to identify already existing community-based women’s organizations (CBOs) that would be willing to participate in the intervention and whose community centers were in close proximity to UNRWA elementary schools.

The CBO in Bourj el Barajneh already had a small functional kitchen that was used for intermittent social events or activities. As for the CBO in Shatila, a meeting room was converted into a kitchen. We renovated the kitchens and equipped them with the necessary items to increase their capacity to produce food on a larger scale and to ensure food safety and hygiene.

The community kitchen intervention involved two components. The first component included a week-long training on-site in the kitchens covering topics related to entrepreneurship (organizational, managerial, purchasing and budgeting skills), food preparation, food safety and hygiene, nutrition, and the development of standardized recipes of healthy school snacks. The training program was developed specifically for the context and implemented in Arabic. The training was tailored for women with low literacy and used visual and practical methods, including strategies for bulk purchasing within the context of Palestinian refugee camps. Training was conducted on-site in the kitchens and involved on-the job skills acquisition. A monthly snack menu was developed by the women using a participatory process including focus group discussions during which women suggested Palestinian dishes that could be made for the school snacks and may appeal to primary-school aged children. The study nutritionist worked with the women to adapt the recipes based on recommended nutrient content for mid-morning school snacks.

The second component provided an employment opportunity by involving women in catering daily healthy school snacks produced in the kitchens, to children aged 5 to 12 years who were attending two UNRWA schools. The school enrollment rate of 7 to12-year-old children generally reaches 97% [1]. Linking the kitchens with schools ensures a market for food produced in the community kitchens. In addition to increased income and wealth creation, another motive to participate in such programs in the refugee context is preserving identity and supporting participants’ displaced ethnic communities [15]. In the *HKHC* intervention, incorporating traditional Palestinian meals in the monthly snack menu was one way of promoting Palestinian culture amongst the younger school generation that eat more non-traditional and fast-food meals.

The intervention involved working in the kitchen for 2 to 3 days a week for 6-h shifts per working day, throughout a period of 8 months (October to June; the duration of the school year). Women who participated arrived at the kitchen early in the day (around 7 am), prepared food and were able to complete all tasks by 1 pm, to be home when their children arrived from school. One of the CBOs had a nursery that provided childcare for pre-school children, the other CBO allowed women to bring young children to the centre to be cared for by CBO staff. During the academic year, the two Healthy Kitchens supplied a healthy mid-morning snack of around 313 kcal, 5 days a week to 714 children attending two UNRWA elementary schools. The snacks were subsidized and schoolchildren were asked to pay 0.25USD per snack, totaling 5USD per month for 20 snacks. Women’s additional income (from snack sales and the subsidy from the program) was equivalent to 110 USD per month.

### Recruitment

With the help of UNRWA’s Relief and Social Services office, Palestinian women living in the camps were identified and contacted by social workers and CBO staff to participate in this intervention. Social workers and CBOs reached out to women who had either applied for the UNRWA social safety net program, or had attended previous CBO activities (language literacy, computer literacy and hairdressing classes), and had previously expressed a need/willingness to work. Fifty one women initially expressed an interest in participating in the intervention and attended an information session about the study. During this session, a detailed explanation of the intervention including time commitment, rotation schedule and monetary compensation was communicated to the women. In addition, written informed consent was sought from all women participants at the beginning of the study. All protocols were approved by the Institutional Review Boards of the American University of Beirut (AUB) and the University of Maryland.

### Evaluation design and data collection

A mixed-methods approach was used to evaluate the intervention. Data were collected by trained staff including UNRWA social workers. Training of data collectors was conducted by the research team at AUB.

#### Quantitative data

Quantitative data were collected using a socio-demographic and economic questionnaire, which included questions on household assets, household income and food expenditure; and for each household member data were collected on employment and educational status. The questions on economic status were administered at baseline and endline to all participating women; one woman did not complete the questionnaire at endline, and therefore data are available on 32 women in total.

Household food insecurity was assessed using the 7-item Arab Family Food Security Scale (AFFSS), previously validated for this population [16]. Positive responses to the questions were summed and households were classified as food secure (score 0–2), moderately food insecure (score 3–5) and severely food insecure (score 6–7). Questions on coping strategies were adapted from the Coping Strategies Index [17].

Decision making power was assessed using several domains adapted from the Women’s Empowerment in Agriculture Index (WEAI) which includes questions on access to and decision-making power over income and expenditures, as well as decisions related to meal planning, healthcare, family planning, and visits to family or relatives [18]. The two components of this questionnaire module asked “When decisions are made regarding the following aspects of household life, who is it that normally takes the decision?”, which enables the collection of responses that indicate sole or join decision making, as well as “To what extent do you feel you can make your own personal decisions regarding these aspects of household life if you want (ed) to?”. Questions related to previous employment history and whether the participant was actively seeking a job were used to assess the motivation of the participant - engagement in more prior activities reflected higher motivation levels.

Respondents were also asked about their health status using the self-rated health question (SRH), with responses ranging from “very good” to “not good at all” [19]. Mental health was assessed using the validated Mental Health Inventory (MHI-5) in Arabic, with higher scores indicating better mental health [20]. The total score was normalized to a 0-100 scale for this analysis. Both SRH and MHI-5 have been previously validated and used in this population [1, 20–22]. The 10-item Duke Social Support Index (DSSI) was translated and used as a continuous measurement to determine the participant’s level of social support [23]. Although this tool has not been validated in this setting specifically, it has been validated and used among several vulnerable groups in various contexts [24–26].

#### Qualitative data

Qualitative data collection entailed semi-structured interviews with the 32 women at the end of the study. Face-to-face interviews were conducted by two trained research assistants not involved in training and implementation of the intervention and lasted between 30 and 60 min each. The interviews were guided by a topic guide that included questions around women’s experience in the community kitchen, interactions with others, financial wellbeing, perceived impact of the intervention, and advantages and disadvantages of the project. The open-ended questions also aimed at capturing in-depth descriptions of the role of the project in the financial and social wellbeing of the women. The interviews were tape-recorded, transcribed, and translated from Arabic to English.

### Data analysis

Baseline differences between the women who dropped out versus those who remained in the study were tested using non-parametric methods (quantitative Wilcoxon ranksum) and Fisher exact tests. To examine the association between paired baseline and endline outcomes, McNemar chi-square test (nominal variables) and Wilcoxon signrank (continuous variables) were used. A *p*-value of 0.05 was used to indicate statistical significance. All analyses were performed using Stata 13 (StataCorp).

Transcripts of interviews were analyzed using thematic analysis in NVivo10 (QSR International). An initial reading of transcripts led to a preliminary list of recurrent themes. Guided by the original research questions and the themes that emerged, we organized the data into categories and added illustrative quotes. All codes were revised by two researchers to check the consistency in the categorization of the text.

## Results

### Characteristics of the study population

A convenience sample of 51 women was initially recruited to the intervention. However, in the first week, when they became aware of the time commitment necessary, 18 women dropped out. One further woman did not complete the endline questionnaire. There were no significant differences in household expenditure, food security and mental health characteristics at baseline between women who remained in the study and those who left the study. However, the women who remained in the study were from larger households (median number of household members 6 [4–7] vs 4 [3–6]) (*p* < 0.05), had a higher social support score (median score 24 [22–25] vs 22 [20–24]) (*p* < 0.05) and higher motivation levels, indicated by previous engagement in income generating activities (16/32 (50.0%) vs 2/18 (11.1%) (*p* < 0.05).

Throughout the 8 months, 32 women participated in the intervention and completed both baseline and endline assessment. Baseline characteristics of these 32 women are presented in Table 1. Median age of participants was 41 years (range 18–64 years), and the majority were married (*n* = 28/32). Few women completed middle school (*n* = 7/32), and their median household expenditure was 815 USD per month (162 USD per capita) at baseline (Table 1).

**Table 1:** Baseline demographic and entrepreneurship characteristics of the 32 participants in the *HKHC* intervention

Baseline characteristics (*n* = 32)	
Demographic	
Age, median [IQR]	41 [35.5–45]
Marital status, married, n (%)	28 (87.5)
Educational achievement, completed middle school, n (%)	7 (21.9)
Head of household, n (%)	7 (21.9)
Worked in the previous week, n (%)	7 (21.9)
Household size, median [IQR]^a^	6 [4–7]
Household working members, number, median [IQR]	2 [1–2]
Entrepreneurship skills	
Intend to engage in new income generating activities, n (%)	28 (87.5)
Currently engaged in income generating activities, n (%)	16 (50.0)

^a^ *[IQR]* Interquartile range

### Household economics

Additional income generated from the intervention was equivalent to a median of 110 USD per month. Total household expenditure increased by 13%, which was due to a significant increase in expenditure on food (*p* < 0.04), clothing (*p* < 0.001) and entertainment (*p* < 0.001) **(**Table 2). There was also an increase, although not significant, in water, transportation, tuition, and healthcare expenditures per capita (data not shown).

**Table 2:** Baseline and endline economic, food security, decision making, mental health and social support indicators of participants

	Baseline (*N* = 32)	Endline (*N* = 32)	*P* value
Economic wellbeing			
Household assets^a^, number, median [IQR]	8 [6.5–9]	8 [7–10]	NS
Household expenditure, total monthly USD per capita, median [IQR]	162.2 [152.5–165.7]	226.4 [168.7–229.1]	0.009
Food expenditure	66.6 [57.1–81.6]	81.6 [50.0–106.3]	0.040
Clothing expenditure	3.0 [0.6–4.0]	6.6 [3.9–10.3]	0.001
Entertainment expenditure	0.0 [0.0–0.0]	0.55 [0–3.8]	0.001
Crowding index (more than 3 household members per sleeping room), n (%)	15 (46.9)	12 (37.5)	NS
Food security			
AFFSS score, median [IQR]	4 [2;5]	2 [0–3]	0.006
Food secure, n (%)	14 (43.8)	19 (59.3)	NS
Moderately food insecure, n (%)	12 (37.5)	8 (25.0)	NS
Severely food insecure, n (%)	6 (18.8)	5 (15.6)	NS
Decision making			
Woman is the sole decision maker regarding:			
Her employment, n (%)	14 (43.8)	17 (53.1)	NS
Preparation of daily meals, n (%)	18 (56.3)	22 (68.8)	NS
Visiting family, n (%)	12 (37.5)	16 (50.0)	NS
Major household expenditures, n (%)	3 (9.4)	13 (40.6)	0.038
Minor household expenditures, n (%)	22 (68.8)	22 (68.8)	NS
Family planning, n (%)	10 (31.3)	10 (31.3)	NS
Seeking healthcare, n (%)	16 (50.0)	19 (59.4)	NS
Taking medication, n (%)	19 (59.4)	22 (68.8)	NS
Reported health			
Self-reported health, median [IQR]	3 [2–4]	4 [3–4]	NS
Mental health inventory score, median [IQR]	40.9 [22.7–72.7]	57.14 [28.6–83.3]	NS
Social support			
Social support score (DSSI), median [IQR]	24 [22–25]	24 [21–25.5]	NS

*[IQR]* Interquartile range, *AFFSS* Arab family food security scale

^a^ Household asset index was constructed as a sum of ownership of vehicle, fridge, freezer, oven, microwave, washing machine, computer, air conditioner, hot water boiler, mp3 player, land line, mobile phone, internet line and satellite subscription

Some respondents (20 women) in qualitative interviews stated that the additional income improved their overall financial status while others felt that the income was minimal (6 women). Moreover, some women (7 women) stated that earning money incentivized them to work. Others (7 women) indicated that the income allowed them, to a certain extent, to be self-sufficient. One woman mentioned, *“I am taking this money. I earned it, I did it, I worked hard to get it so that was something very nice to me that I became productive”.* Another woman stated, *“But I started feeling that I am productive in something, even if I [just use the money to] recharge [credit] to my phone. If I get my daughter shoes, or glasses, or if I get myself a watch, I started feeling that I am doing something, something from my own effort, something that has a value” [W2].*

Working helped women to no longer view themselves as an economic burden on their husbands; the intervention allowed them to diverge from societal norms which impose that women are completely dependent on their husbands for money. When talking about her colleague in the kitchen, one woman mentioned, *“her husband used to give her money for the house expenses, [now] she is [contributing to paying off] the loan without the constant worry” [W4].*

Moreover, some women stated that the income earned contributed to paying off debt (2 women), while others used it as extra money to buy clothes for children (4 women) and for the healthcare of sick family members (4 women). Income earned was also used towards their children’s education (3 women). One woman recounted, “*I have an afternoon support teacher for my kid. I used to worry about how I was going to cover [the cost] at the end of the month. Now even if the money [amount] we get is small, I put it towards the teacher [salary]. It helped me. It helped me even it was small [amount]” [W24].*

One woman described the project as a means to provide financial stability; *“There is a proverb that says, “a pebble stabilizes a big jar”. This [work] is the pebble that stabilized the big jar. In summary, it is helpful. Thank God, yes, it is true that the amount [salary] is not big but, it closed a big gap” [W13]*.

### Food security, nutrition knowledge and behavior change

At baseline, 12/32 (37.5%) of the women reported that their households experienced moderate food insecurity and 6/32 (18.7%) reported severe food insecurity. This was somewhat reduced at endline to 8/32 (25.0%) moderately food insecure and 5/32 (15.6%) severely food insecure (Table 2). Median AFFSS score went from 4 [2; 5] at baseline to 2 [0; 3] at endline (*p* < 0.01), which was reflected in a reduction in food-related coping strategies and accepting gifts (*p* < 0.01) and a borderline significant decrease in borrowing money to obtain food (*p* = 0.070) and borrowing food (*p* = 0.063). There were no significant differences in household diet diversity between baseline and endline.

However, several women reported changes in food preparation behaviors (23 women). They mentioned the fact that they reduced salt, butter, oil, and fried food consumption at home. One woman said, *“I used to put a lot of salt [in my cooking] so I started lessening it, my hand was loose with the salt, now I put less” [W2].*

The women also highlighted that they implemented the food safety practices they learned in the kitchens to their homes. Their favorite topics were mostly related to hygiene: *“For example the vegetables [cutting] board, you can’t use it for meat. I am now doing this at home. I now have more boards for the vegetables, for the onions, for the meat” [W57].* Overall, the participants were pleased that the information learned had an impact on their daily lives, as food is a central part of their responsibilities at home. Participants mentioned that they improved their cooking skills and learned new recipes that they now prepared at home.

### Decision making, skills acquisition and personal growth

The women reported a shift in their own decision-making power regarding major household expenditures from 3/32 (9.4%) at baseline compared to 13/32 (40.6%) at endline (*p* < 0.05) And although not significant, we observed an increase in the extent to which women felt they could make their own decisions regarding employment, daily meal preparation, major, minor household expenditures and getting advice on healthcare (Table 2).

Women also expressed that the skills gained throughout the training taught them to calculate expenses and scale up kitchen operations (9 women). One woman reported, *“I didn’t know how to calculate the amounts. I used to spend a whole day deciding. And some days we used to get more ingredients. [now] I know how much I need to order. I can tell you the price right away. And I know right away the stuff I need to purchase” [W4].* They expressed satisfaction with learning new tactics on how to shop for groceries efficiently and be more economical in their work.

On a personal level, women reported overcoming shyness, being more responsible, and feeling efficient. The women expressed participating in the kitchen also made them feel valuable (20 women), with one woman stating, [W11] *“my personality really got stronger, before I used to say I’m living, and my kids are what are important. Now, I want to live and to prove [show] that I exist [and] I have my personality. Just like a man can work and be productive, a woman as well can work and be productive and the rights should be equal between a man and a woman. Now [ …*] *I feel that I am a productive woman.” [W7].*

All 32 women also perceived growth within the group where they learned to work as a team, how to delegate tasks appropriately, and to collectively encourage each other. One woman noted, *“the project got me out of the house. [It] introduced us to new people, [ …*] *to a healthy eating program [ …*] *to how to cooperate within a group, to get integrated, to respect each other. We learned a lot of things. The work became group work. For example, when I am rolling grape leaves at home, I get bored alone. When we are a group, you feel that one woman encourages the other. This way we want to do more” [W24].*

The women were also seen as key players in the school feeding program which gave them a sense of accomplishment. Several women embraced the fact that they were able to provide for themselves while providing for others and serving the community (8 women). They received positive feedback on the quality of the food and community members were interested in their work: *“All my neighbors are fans, when I show up, they ask me “what did you cook today?”, so I tell them, there are things they don’t know so I explain” [W70].*

### Mental health and social support

Data from the quantitative component of the study showed a non-significant increase in MHI-5 score, and no significant difference in the social support scale (Table 2). 21/32 women had some level of increase in MHI-5 score between baseline and endline, whereas 11/32 women had a decrease.

However, many of the women highlighted in the interview that the kitchen provided an escape from their everyday lives, whether it was to temporarily distance themselves from family, from the environment at home, or as a break from their daily routine (15 women). Several women noted that they saw value in being occupied with their work and felt more energetic throughout their day in contrast to their otherwise sedentary lifestyle (15 women). Many women stressed that their work in the kitchen was a source of happiness, boosted their morale, confidence, self-worth, and reduced anxiety (20 women).

In addition, women expressed that the kitchen provided additional social support (28 women). Throughout their work in the kitchen, the women stepped out of their comfort zone, shared problems with each other, and valued teamwork: *“We are very collaborative; this is why we feel comfortable. For example, if there’s a pile of dishes to wash, you don’t have to tell someone to do it, she would come on her own and wash them” [W68].* The women felt they gained a sense of friendship from working in the Healthy Kitchens. This happened through overcoming social isolation and emotionally supporting each other in a hospitable environment: *“You feel all women are united, there’s no gossiping, you feel that we’re all sisters sitting in one big house and cooking for our families. The experience I lived was great” [W62].*

Women reported learning how to listen to each other and communicate effectively. One woman indicated, *“so at home, each woman would cook her way and give orders to her family. Then, we progressively learned to talk to each other without being sensitive [ …*]” *. [W44].*

### Investigating the discrepancy

In order to explore the discrepancy observed between the null quantitative results on mental health and social support with the generally positive qualitative findings, we disaggregated both quantitative and qualitative data by MHI5-score change (Comparing women whose MHI-5 score decreased between baseline and endline with women whose scores increased).

**Fig. 1:**
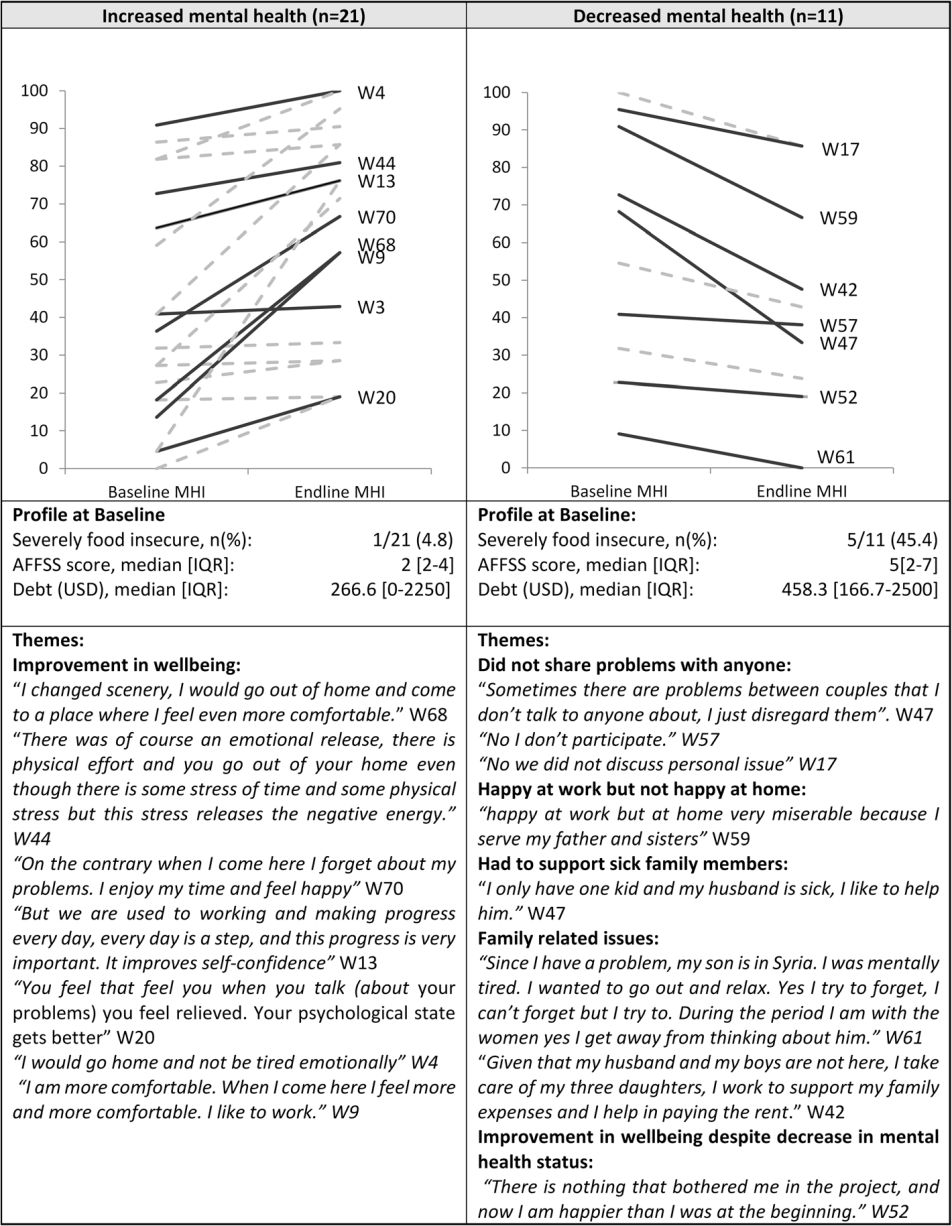
Changes in mental health inventory score and themes associated with the change

Women with decreased mental health scores were more likely to be have been severely food insecure at baseline (*p* < 0.01) and had higher debt (not statistically significant) than the women who had an improvement in mental health scores (Fig. 1).

Those who exhibited the sharpest decrease in mental health referred to a set of underlying circumstances that affected their mental health, such as choosing not to share problems with anyone, expressing being satisfied at work but not at home because of family problems and family breakups, and the need to support sick family members (Fig. 1).

## Discussion

This study provides evidence of the potential of the *HKHC* model to improve economic status, food security and entrepreneurial skills of women living in marginalized low-income communities as well as providing a place for social interaction.

The integration of an income-generating component into community kitchens transformed these into social enterprises, allowing women living in this traditionally patriarchal community to “enact agency within the context of constraint” [27–29]. That is, in Palestinian refugee communities, as in many Arab countries, societal norms limit the sectors in which women can work and may require them to obtain permission from their husbands or fathers to seek employment [30, 31]. Where formal employment is limited [1, 31], particularly among married women, home-based self-employment has been argued to provide a safe environment from which women can challenge their traditional roles [27, 29], and allow them to become productive household members. The *HKHC* model broadens the concept of home-based entrepreneurship, by applying it at the community level, where, along with creating income generating potential, it built a sense of social support, while not challenging traditional social norms [32, 33].

In fact, women who participated in the intervention increased their spending, self-sufficiency, and decision-making around this spending, ranging from minor expenditures such as clothing and entertainment to major expenditures such as debt repayments, in spite of sociocultural barriers. Working and earning money provided participants with a sense of self-satisfaction [27].

Although the income generated was modest, participants’ household food security significantly improved from baseline. In low-income populations, women are especially vulnerable to food insecurity [34]. Studies examining the impact of food-based interventions on empowerment of women in the household and on their food security status showed the importance of including women as active players in solutions to address household food insecurity [35, 36]. In our study, there was a significant increase in women’s own decision-making power regarding major household expenditure and, although non-significant, there was an increase in women making their own decisions regarding daily meal preparation. Evidence from Africa, Asia and Latin America shows that women’s access to income, or an increase in household decision-making regarding expenditure, is associated with improvements in household food security [37]. This is partly due to women spending a significantly higher proportion of their income compared to men on food and health-related issues [37] and to their role in food production, food preparation and childcare [34, 38].

The *HKHC* intervention provided the women with knowledge on how to improve food security, in addition to some financial means to access food through generated income. Socioeconomic status has been shown to be positively and significantly associated with food security in the Palestinian refugee population and in other vulnerable populations [1, 39]. The significant increase in food expenditure coupled with the nutrition training component of the intervention may indicate improved access to food and knowledge and therefore, improved food security. We did not measure household food consumption and could not examine the impact of improved food security on food choices. This may be of interest in future studies.

Studies have shown that women’s mental health is strongly associated with food security [34, 40, 41]. In addition, social support can be obtained through the workplace and is associated with better physical and mental health [42] and improved job satisfaction [43]. The *HKHC* intervention thus attempted to improve mental health by addressing food security and increasing the social network of women, however although the majority of women who participated in the study had an increase in mental health score, these were not statistically significant increases. Participants indicated the importance of work in giving them a break from their daily routine and providing them with an opportunity to leave the house and interact with other women. Participants commented on their feelings of satisfaction with the work they were doing and in the team approach to their work. They reported that the kitchen provided a space and opportunity to share their problems with one another and bring women out of their isolation, these results align with those of another study [11]. In most developing countries, the informal work sector is the primary source of employment for women, with the vast majority of these women being home-based workers [44]. Although regional data are scarce, one study suggests that the majority of working women in Jordan are self-employed and operate from their homes [28, 45]. Home-based workers have been shown to be less likely to develop social ties outside the family compared to those working outside of the home. Also, home-based workers may be at higher risk of poverty compared to individuals in formal wage employment [46]. The *HKHC* intervention allowed women to participate in a safe environment within the formal work sector, using a community-based approach to expand on the concept of “safe” work environment.

Despite many positive comments from women, one third of participants did not show improvement in their mental health and in fact, some showed a decline. We attempted to further investigate the reason behind these discrepancies by comparing characteristics of women who had improved mental health scores with those who did not. Our data showed that the presence of an underlying stressful condition, not addressed in this study, linked to conflict and health complexities, was among the key barriers to changes in mental health conditions of these participants. These women, however, continued to work and some expressed positive feelings towards the project despite hardships they were facing in their home lives.

At the onset of the project more women signed up to participate in the project, however, 18 dropped out within the first week. Due to time constraints, employment affects women’s ability to provide childcare and perform their other household chores such as cooking. In fact, the main reasons for not working reported by women ages 25–54 include housework and other family reasons [31]. Our study took this into consideration in the design of the *HKHC* model, which was informed by focus groups conducted with women at the onset of the intervention when schedules and time commitments were discussed. The intervention was designed to avoid major changes in their existing routines and the CBOs provided options for preschool childcare. Despite these efforts, the women who remained in the study had better social support compared to those who dropped out. Studies have shown that family support can provide emotional and instrumental support to working women [47]. Also, support towards childcare has a positive impact on preventing work-family conflict [48, 49], and may reduce perceptions of formal employment interfering negatively with domestic responsibilities [50]. In societies with more traditional gender roles, the redistribution of power within the household and active participation from men in childcare responsibilities is required to facilitate women’s ability to work [51].

The main limitation of this study lies in the fact that there was no counterfactual (control group that did not receive the intervention), and we cannot therefore directly attribute the outcomes measured in the study to the intervention. The quantitative results are also limited by the small sample size which may not have allowed us to detect significant changes in mental health and social support, among other variables. The high drop-out rates of women at the beginning of the intervention reflect the challenges for women to participate in such programs. The women who remained in the study had higher social support and more work-experience which indicate that in this society, social support is essential in order for women to be able to work outside the home. However, the qualitative data provided a depth of knowledge that supplemented our quantitative data. Although it is possible that responses in the interviews were influenced by social desirability bias, we employed interviewers that had not been part of the intervention team, and assured anonymity in the research process to minimize this to the extent possible. Another limitation is that changes in decision making roles and other domains of empowerment can entail a long-term process. The duration of the study may therefore not have been long enough to incur changes of a longer-term nature.

The two CBOs have now established themselves as catering businesses and are sustaining their operation by providing food for schools, a pre-school and an orphanage, as well as catering for local events (Ramadan dinners for older adults, festivals). Future studies would benefit from longer-term follow up to assess sustained differences in participants’ lives, in addition to considering quasi-experimental or randomized designs with larger sample sizes to enable the assessment of effectiveness of the intervention.

## Conclusion

The findings from this study lend insight into the design and potential of community kitchens among low-income, food insecure populations; refugee or migrant populations; and women with limited formal work experience. The HKHC model created a social enterprise using the concept of community kitchens linked to schools and allowed women to significantly contribute to household expenditure and improve their food security. Results highlight the importance of using a multi-sectoral approach to address the social determinants of food insecurity in vulnerable women living in chronic political and economic constraints.

Community kitchens are increasingly being used in high-income countries (the United States for example), with the aim to provide professional development, supplementary income, and a flexible, safe work environment for low-income or refugee/migrant women who wish to enter the workforce. As refugee resettlement and migration continue to rise, the *HKHC* intervention outlines a roadmap for how such a model could provide women a route into the formal workforce using their skills as talented home cooks, build on that skillset in a safe environment, contribute to poverty reduction – which is at the heart of food insecurity - and provide a supportive network.

## Data Availability

The datasets generated and/or analysed during the current study are not publicly available due to ethical restrictions but are available from the corresponding author on reasonable request.

## Abbreviations

AFFSS: Arab Family Food Security Scale
AUB: American University of Beirut
CBO: Community-based organization
DSSI: Duke Social Support Index
HKHC: Healthy Kitchens, Healthy Children
IQR: Interquartile range
MHI-5: Mental Health Inventory
SRH: Self-rated health
UNRWA: UN Relief and Works Agency for Palestine refugees
WEAI: Women’s Empowerment in Agriculture Index
